# Sutured tendon repair; a multi-scale finite element model

**DOI:** 10.1007/s10237-014-0593-5

**Published:** 2014-05-20

**Authors:** Shelley D. Rawson, Lee Margetts, Jason K. F. Wong, Sarah H. Cartmell

**Affiliations:** 1 E12, Materials Science Centre, University of Manchester, Oxford Road, Grosvenor Street, Manchester, M1 7HS UK; 2 School of Earth, Atmospheric and Environmental Sciences, University of Manchester, Oxford Road, Manchester, M13 9PL UK; 3 Plastic Surgery Research, Stopford Building, University of Manchester, Oxford Road, Manchester, M13 9PT UK

**Keywords:** Finite element modelling, Tendon, Homogenisation, Kessler, Multi-scale modelling, Suture

## Abstract

Following rupture, tendons are sutured to reapproximate the severed ends and permit healing. Several repair techniques are employed clinically, with recent focus towards high-strength sutures, permitting early active mobilisation thus improving resultant joint mobility. However, the arrangement of suture repairs locally alters the loading environment experienced by the tendon. The extent of the augmented stress distribution and its effect on the tissue is unknown. Stress distribution cannot be established using traditional tensile testing, in vivo, or ex vivo study of suture repairs. We have developed a 3D finite element model of a Kessler suture repair employing multiscale modelling to represent tendon microstructure and incorporate its highly orthotropic behaviour into the tissue description. This was informed by ex vivo tensile testing of porcine flexor digitorum profundus tendon. The transverse modulus of the tendon was 0.2551 $\pm$ 0.0818 MPa and 0.1035 $\pm$ 0.0454 MPa in proximal and distal tendon samples, respectively, and the interfibrillar tissue modulus ranged from 0.1021 to 0.0416 MPa. We observed an elliptically shaped region of high stress around the suture anchor, consistent with a known region of acellularity which develop 72 h post-operatively and remain for at least a year. We also observed a stress shielded region close to the severed tendon ends, which may impair collagen fibre realignment during the remodelling stage of repair due to the lack of tensile stress.

## Introduction

Tendon injury affects approximately 50/100,000 people in the UK per annum resulting from laceration, crush, sporting injuries or degenerative disorders (Clayton and Court-Brown 2008). Hand flexor tendon injury usually results from laceration or crush damage and despite a century of innovation, 25 % of these patients experience a deficit in mobility following tendon repair (Su et al. 2005). Repair is achieved by suturing the defect to rejoin the severed ends, and many suture arrangements have been proposed (Wu and Tang 2013). The current trend is towards strong repairs to permit early active mobilisation which is beneficial to regaining functional motion of the tendon. High strength is achieved by employing locking suture loops to grip onto fibre bundles (Pennington 1979) and by increasing the number of suture strands crossing the defect (Savage 1985). Whilst this benefits strength, the detriment to the tissue due to the presence and arrangement of the suture has received little attention. Since absorbable suture is not favoured (Trail et al. 1989), the effects of suture presence on the tissue are of interest for long-term tissue health.

The failure of the commonly used modified Kessler suture was imaged using X-ray in human cadaver digital flexor tendons, showing total collapse of the locking loops during loading. This demonstrates that locking loops can cause tissue damage, even within the acceptable operating range of 2 mm gapping (Mashadi and Amis 1991). Wong et al. describes an acellular zone due to cell necrosis when suture and tension are present, arising within 30 min of suturing and remaining at one year post-operatively (Wong et al. 2010). It was hypothesised that the acellular zone arose due to stresses in the tissue. Fibroblasts synthesise collagen and contribute to the mechanical integrity of tendon (Galloway et al. 2013), thus acellular regions are detrimental to both healing and functionality. In light of these studies, the suitability of current suture repairs in providing a mechanically favourable environment for the tissue is questionable.

Acellular tendon regions have been observed in the Kessler suture repair (Wong et al. 2010). Identification of similar acellular regions within the various suture arrangements would aid the surgeon in selecting a repair which provides both mechanical stability and a favourable environment for healing. An in silico model, validated against observations of Wong et al., could be adapted to study the tissue insult resulting from the variety of suture repairs. This will aid suture technique selection for healing and long-term tissue functionality and may be adapted for the prototyping and validation of new repair methods. Benefits of in silico validation include rapid prototyping, reduced cost, and reduced use of animals in line with 3R’s guidelines (NC3Rs 2012).

To produce an in silico model of a suture repair, tendon mechanical behaviour must be defined, which is largely influenced by tendon structure. Tendon dry weight is 68.5–88 % type 1 collagen (Koob and Vogel 1987), which self assembles into long supermolecular structures called fibrils (Fraser et al. 1983) whose tensile modulus is 200 to 3,000 MPa as determined by atomic force microscopy (Rijt et al. 2006; Svensson et al. 2010). Fibrils are arranged in aligned fibres, which are in turn arranged into fascicles which form the tendon (Strickland 2005). This highly aligned structure results in an orthotropic material, whose tensile modulus is 350 to 850 MPa in the longitudinal direction as demonstrated by ex vivo tensile testing (Butler et al. 1984; Wren et al. 2001). Transverse tendon modulus has received little attention; human cadaver supraspinatus tendon was found to have a transverse modulus of 1 to 40 MPa (Lake et al. 2010); however, a modulus of 0.157 MPa was obtained during transverse tensile testing of ovine flexor tendons (Lynch et al. 2003). Variation in results may be due to differing anatomical locations and species. The present work employed porcine flexor digitorum profundus (FDP) tendon, a suitable surrogate for human tendon (Havulinna et al. 2011), to determine the transverse modulus and fully define the orthotropic behaviour of the tissue.

Finite element modelling (FEM) has been employed to produce an in silico model of a suture repaired tendon. Since the 1970s, finite element analysis (FEA) has been employed for the study of biomechanics, and tendon tissue has been modelled in a variety of ways. Tendon geometry is commonly simplified to 1D (García-González et al. 2009) or 2D (Wakabayashi et al. 2003) structures in cases where other interacting tissues are the focus of the study, or where the simplification does not impede results. The material description of tendon is also subject to simplification and is commonly described as isotropic linear elastic. Tendon has also been modelled akin to a fibre reinforced composite; modelling the fibrils and surrounding tissue to study crimp formation (Herchenhan et al. 2012) and the effects of microstructure on Poisson’s ratio (Reese et al. 2010).

The aim of the present work is to observe the stress and deformation imposed on the tendon tissue by suture when a force is applied, simulating rehabilitation of the repaired tendon. Finite element (FE) analysis was employed to model a sutured tendon repair subject to tension. The FE model was validated against laboratory results. As part of this study, it was necessary to employ 3D modelling and an orthotropic description of tendon tissue since the suture techniques rely on the anisotropy of tendon and strength of the fibrils to provide repair strength. It was impractical to model all the individual fibrils within the suture repair model due to computational cost. As such, a separate model of tendon microstructure was employed, detailing the fibrils and surrounding tissue. Homogenisation was then performed on the microstructure model, and the resultant orthotropic material description was input into the suture repair model. This is a novel approach for the observation of stress and deformation in suture repaired tendons, not published before in the literature. We hypothesise that acellular regions highlighted in previous studies correlate with regions of high stress in the tissue during loading (Wong et al. 2010).

## Methods

### Obtaining tendon samples

Tendons were obtained from porcine fore trotters sourced from a local abattoir less than 24 h after slaughter. The porcine FDP tendon, which is a suitable surrogate for human FDP tendon (Havulinna et al. 2011), was extracted from the central two toes of the trotters and stored in PBS at room temperature until testing, which was performed the same day.

### Tendon transverse mechanical behaviour

**Fig. 1:**
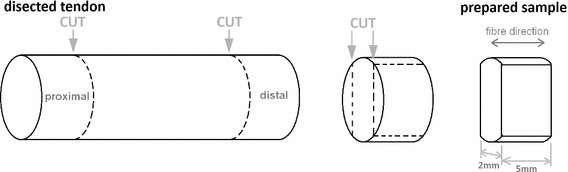
Preparation of tendon samples for transverse tensile testing

**Fig. 2:**
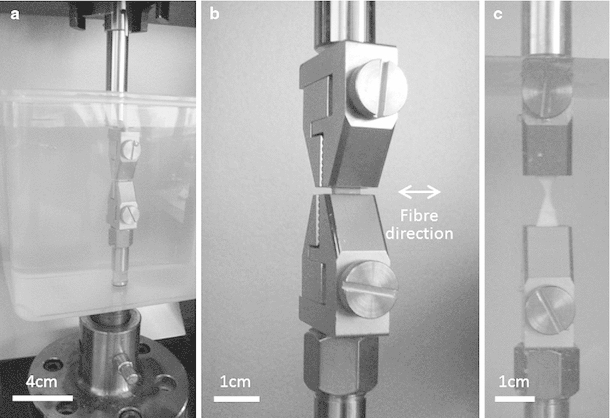
Tendon loaded in tissue grips prior to transverse tensile testing. **a** Full apparatus including PBS filled tank. **b** Sample prior to loading, shown in grips without tank for clarity. **c** Sample after failure

Proximal and distal samples were dissected from the harvested tendons, cut to 5 mm wide and 2 mm thick using a scalpel and measured using digital vernier callipers (n = 12; Fig. 1). Sample length was equal to tendon thickness. A rectangular cross section was desired for ease of gripping, removal of the epitenon and to enable calculation of stress and strain. An Instron 5,569 load frame (Instron, High Wycombe, UK) with a 100N load cell, adapted to fit Bose serrated tissue grips (Bose Corporation, Eden Prarie, Minnesota, USA) was employed for tensile testing. Tissue grips were placed 2.5 mm apart prior to gripping of each sample, ensuring a consistent initial testing length. No pretension was applied due to the low loads at which the sample failed. Compliance in the system was negligible and thus not accounted for. Samples were tested to failure whilst submerged in Dulbecco’s Phosphate Buffer Solution (without Ca2+ or Mg2+; PBS; PAA Laboratories, Pasching, Austria) at a strain rate of 1 and 10 %/s which represents strain rates during quasi-static conditions and normal daily activity, respectively (Wren et al. 2001; Lewis and Shaw 1997) (Fig. 2). The modulus of the approximately linear region of the stress strain curve was obtained before calculation of the mean.

### Calculating matrix modulus

The rule of mixtures was employed to calculate matrix modulus (Eq. 1) (Callister and Rethwisch 2011). Accounting for tissue variation, the fibril and matrix modulus were defined as a range rather than an absolute value. Fibril volume fraction (Vf) was 0.6 (Reese et al. 2010), and the lower and upper limits of fibril modulus (Ef) were 200 and 3,000 MPa, respectively, as defined in literature (Rijt et al. 2006; Svensson et al. 2010). Transverse modulus (ECT) was obtained experimentally (Sect. 2.2), and the lower and upper matrix modulus values (Em) were calculated using Eq. 1. To calculate the lower modulus limit, Ef was assigned the value of 200 MPa and the lower ECT value was used. To calculate the upper modulus limit, Ef was assigned the value of 3,000 MPa and the upper ECT value was used.

1 $$\begin{aligned} E_\mathrm{CT} =\frac{E_{m} E_{f}}{\left( {1-V_{f}} \right) E_{f} +V_{f} E_{m}} \end{aligned}$$

### Finite element model of tendon microstructure

**Fig. 3:**
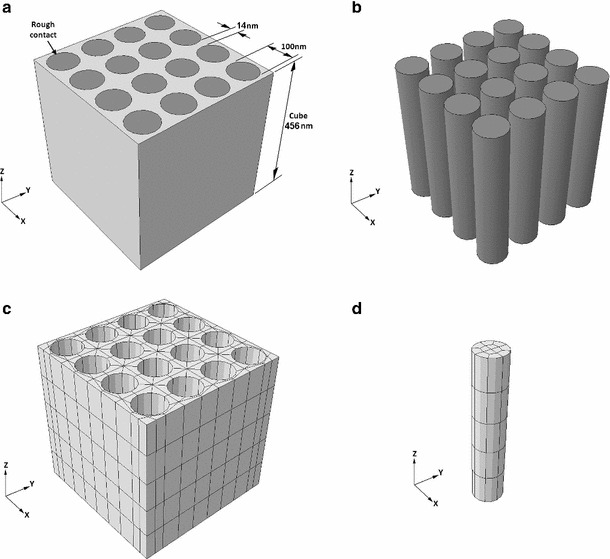
Microstructure finite element model showing geometry of fibrils and matrix (**a** and **b**) and Mesh of matrix (**c**) and fibrils (**d**)

Tendon microstructure was assumed to behave akin to a continuous fibre reinforced composite, whose fibres were the fibrils, and whose matrix was the surrounding tissue. An idealised FE model of one repeating unit cell was produced using Abaqus (version 6.10; Simulia, Providence, Rhode Island, USA). Fibril volume fraction of approximately 0.6 was employed as described by Reese et al. (2010), and fibril diameter was set to 100 nm, midway between the range of fibril sizes found by Baek et al. (1998) resulting in fibril spacing of 14 nm (Fig. 3). The dimensions of the repeating unit cell were chosen to be large enough to minimise the stress inconsistencies at the edges whilst still allowing an appropriately sized mesh and not compromising on computational cost. Rough contact was assigned between the fibrils and matrix, and surfaces were not permitted to separate. A mesh of 3D hexahedron quadratic elements with reduced integration (C3D20R) elements was employed with 820 elements in the matrix and 60 elements in each fibril.

Fibrils and matrix were both defined as isotropic linear elastic materials with a Poisson’s ratio of 0.3. A fibril modulus of 1,700 MPa was chosen and maintained for all models, as this is within the range or Young’s modulus values given in literature (Rijt et al. 2006; Svensson et al. 2010). Matrix modulus was initially set to 1,700 MPa to validate the authors’ implementation of the homogenisation method, checking that homogenising mathematically a composite comprising two materials with the same modulus (1,700 MPa) gave a homogenised material with identical modulus (1,700 MPa).

The matrix modulus was then iteratively reduced by an order of magnitude, approaching the value observed experimentally. FE software suffers numerical difficulties (unable to find a solution) when the model incorporates two materials of very different modulus. The first attempt at analysis with the actual experimental values failed due to this issue. The strategy adopted here brought the numerical values used in the FEA as close as possible to the experimental ones. This methodology is broadly accepted in engineering simulation and has been used elsewhere in biomechanics (Herchenhan et al. 2012).

Once the modulus values were determined, homogenisation was performed; two tensile and two shear strains were applied to the microstructure model, the resultant stresses in each direction were obtained and the engineering constants were computed. The engineering constants (the tangential moduli; E1, E2, E3, the Poisson’s ratios; $\nu$12, $\nu$13, $\nu$23, and the shear moduli; G12, G13, G23) describe the homogenised orthotropic material behaviour that is representative of the microstructure. This process is detailed in Table 1. Had the material been fully orthotropic, additional tensile and shear analyses would have been necessary to obtain all the engineering constants; however, since tendon is transversely isotropic, some of the tests are effectively identical and would therefore yield the same results.

**Table 1:**
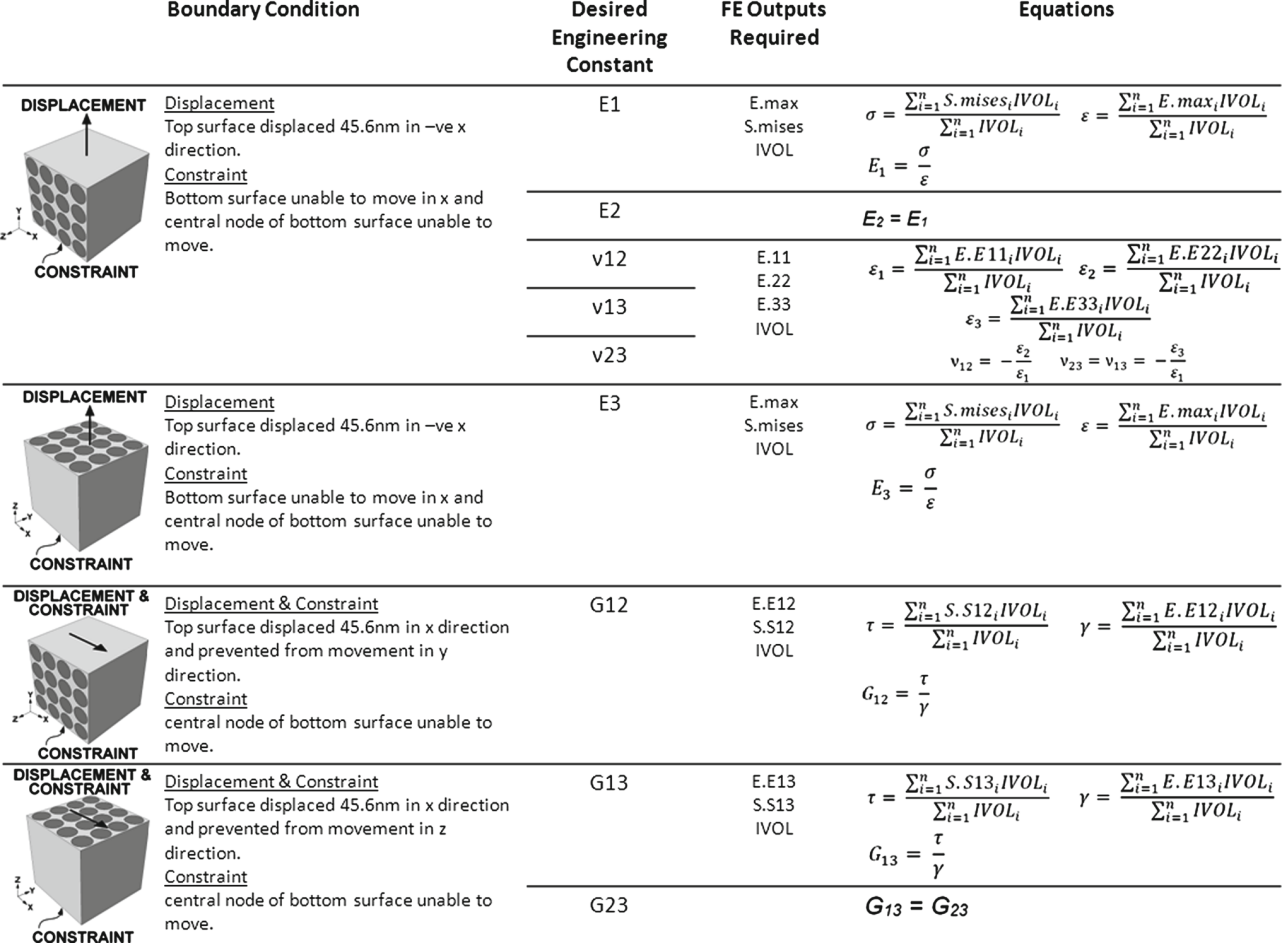
Homogenisation of the microstructure model using FEA to obtain engineering constants

En = tangential modulus in the n, direction, $\nu$nm = shear moduli mn direction, Gmn = shear modulus in the mn direction. All FE outputs are describing behaviour of the integration points, E.max = maximum principal strain, S.mises = Von Mises stress, IVOL = integration point volume, E.Emn = Strain in mn direction, S.Smn = strain mn direction, $\sigma$ = stress in whole volume, $\varepsilon$ = strain in whole volume, $\tau$ = shear stress in whole volume, $\gamma$ = shear strain in whole volume

### Laboratory Kessler tensile testing

**Fig. 4:**
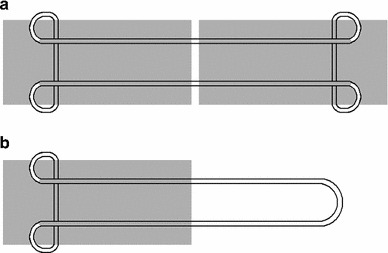
Schematic of **a** Kessler suture repair, and **b** half Kessler repair (*Grey* = tendon. *White* and *black* line = suture external of tendon. *Grey* and *black* line = suture internal of tendon)

**Fig. 5:**
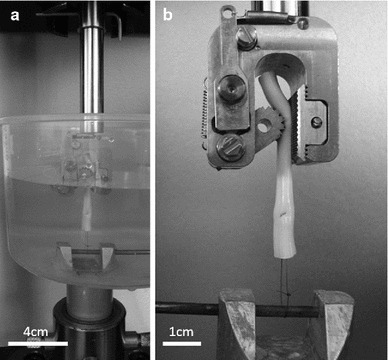
Half Kessler sutured tendon in Bose tissue grip and custom apparatus prior to tensile testing. **a** Full apparatus including PBS filled tank. **b** Sample prior to loading, shown in grips without tank for clarity

Tensile tests were performed on full and half repairs (Fig. 4a and b respectively), and no significant difference in tensile strength was observed (data not shown) due to symmetry in the loading arrangement. Half Kessler suture repairs were performed in the tendon samples using 4–0 Prolene suture (Ethicon Ltd, Edinburgh, UK) ensuring a consistent repair length of 1 cm (Fig. 4b) (n = 12). Samples were tested in PBS at a strain rate of 1 %/s using an Instron 5569 load frame (Instron, High Wycombe, UK) adapted to fit a Bose biodynamic tissue grip (Bose Corporation, Eden Prarie, Minnesota, USA) and a custom grip. The tissue grip held the tendon end, and the suture was looped around the custom grip (Fig. 5). A pretension of 0.1N wasapplied to ensure the sample was taut prior to testing. Compliance of the system was assessed by performing a tensile test with a loop of suture spanning the grips. Compliance within the system was subtracted from tensile test results and the mean force with increasing displacement was calculated.

### Kessler finite element model

**Fig. 6:**
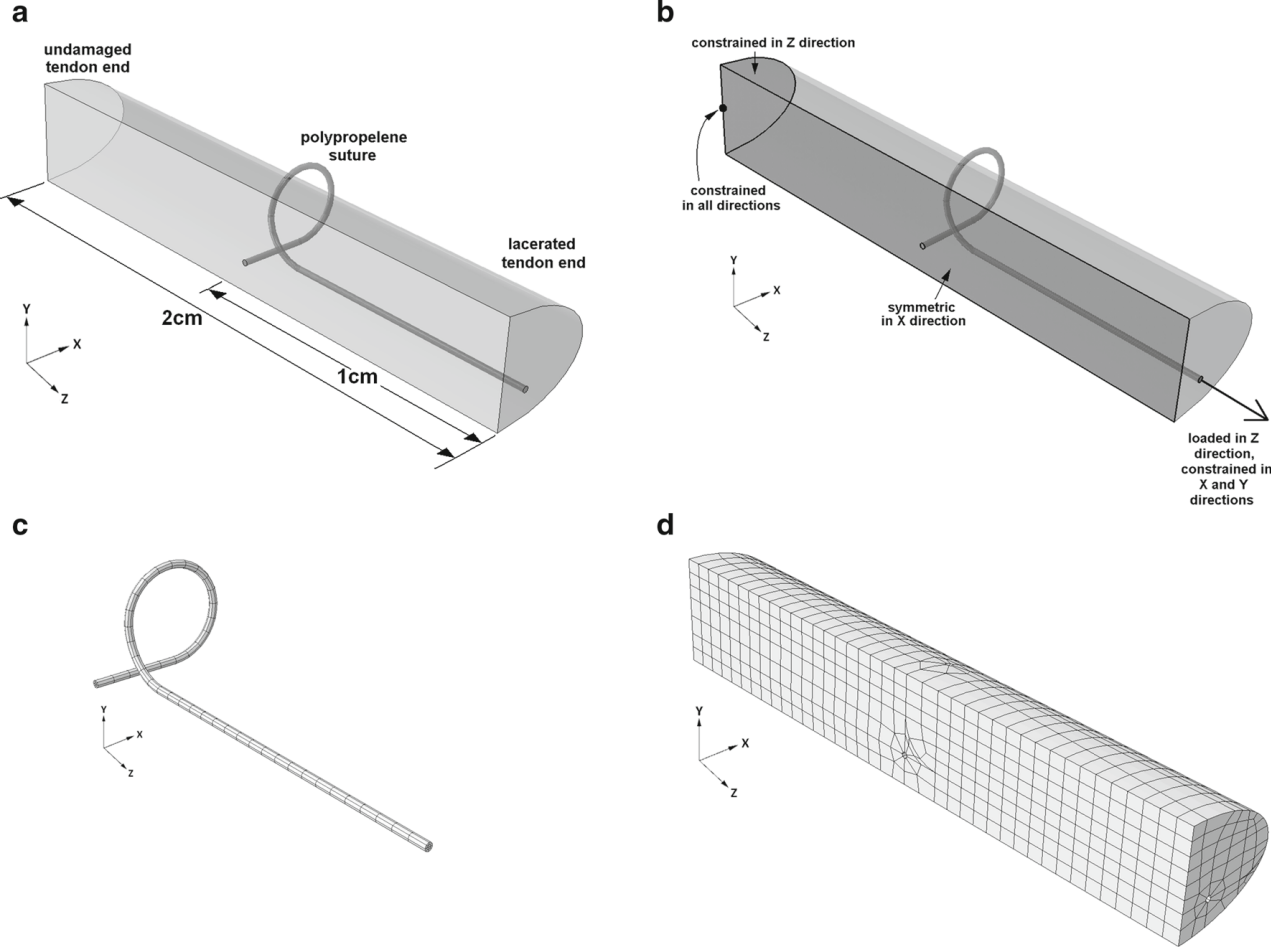
Finite element model of Kessler suture repaired tendon. **a** Arrangement of model. **b** Boundary conditions and load. **c** Suture mesh. **d** Tendon mesh

A grasping Kessler suture arrangement, (Fig. 4a) was modelled using Abaqus (version 6.10; Simulia, Providence, Rhode Island, USA). The thickness and width of the modelled tendon were 3.5 and 6.5 mm, respectively, following digital vernier calliper measurement of 10 fresh porcine FDP tendons. Due to symmetry, it was only necessary to construct one quarter of the repair; stress and displacement in the un-modelled region is a mirror image of the results obtained. Suture diameter was 0.2 mm, consistent with the 4–0 Prolene suture used during tensile testing. Three boundary conditions were applied to the tendon as shown in Fig. 6b. A friction coefficient of 0.005 was assigned between the suture and tendon. A mesh of C3D20R elements was employed with 540 elements in the suture and 1963 elements in the tendon (Fig. 6c and d respectively). The initial state of the suture repair model simulates contact between the tendon ends.

The suture was described as a homogenous linear elastic material with Poisson’s ratio of 0.4 and Young’s modulus of 1 GPa (Callister and Rethwisch 2011). Isotropic and orthotropic linear elastic descriptions were both employed to describe tendon tissue. For the isotropic model, a Poisson’s ration of 0.4 was employed, and Young’s modulus was varied between 2,000 and 0.2 MPa. Engineering constants were used to describe the orthotropic model, obtained following homogenisation of the microstructure model.

**Fig. 7:**
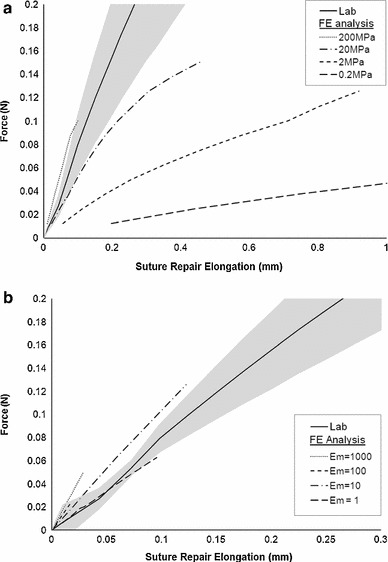
Half Kessler suture repair, comparing lab results (*solid line*, showing standard error as the *grey shaded* region) and finite element analysis results (**a**) using an isotropic linear elastic material description with Poisson’s ratio of 0.4 and varying tendon modulus. **b** using an orthotropic linear elastic material description with Poisson’s ratio of 0.3, fibril modulus of 1,700 MPa and varying matrix modulus

Increments of load were applied from zero up to a maximum to generate load-displacement curves for comparison with the experimental results (Fig. 7). The curves end at the point where Abaqus had difficulties with solving the system. This arises when some of the FEs become too distorted. A workaround is to continuously remesh the models, but this additional computational effort was not thought to add to the insight. The maximum values of loads achieved follow. A maximum load of 0.1005N was applied to the FE isotropic model, and maximum loads of 0.0251, 0.1257, and 0.0628 N were applied to the FE orthotropic models when matrix modulus was set to 100, 10 and 1 MPa, respectively. A 0.5 mm gap between tendon ends was simulated to observe high-stress regions in the tendon tissue.

## Results

### Tendon transverse modulus, matrix modulus and orthotropic description

Transverse modulus was independent of strain rate when varied between 1 and 10 %/s. The mean modulus of distal and proximal samples was 0.1035 $\pm$ 0.0454 MPa and 0.2551 $\pm$ 0.0818 MPa, respectively. Employing the rule of mixtures (Eq. 1), the upper and lower limits of matrix modulus (Em) were calculated as 0.1021 and 0.0416 MPa, respectively.

Following homogenisation of the microstructure FE model, the engineering constants are noted in Table 2 whereby direction 3 is parallel with the fibrils. When fibril and matrix modulus were both 1,700 MPa, results agree with expected values, validating the homogenisation method. It was not possible to obtain homogenisation results when the matrix is reduced to 0.1 MPa due to the large difference between the fibril and matrix modulus.

**Table 2:** Combinations of fibril modulus, matrix modulus and Poisson’s ratio used in microstructure model and resultant engineering constants obtained following homogenisation

Poisson’s Ratio	0.3	0.3	0.3	0.3	0.3
Fibril modulus (MPa)	1,700	1,700	1,700	1,700	1,700
Matrix modulus (MPa)	1,700	1,000	100	10	1
$\hbox {E}_{1}$ (MPa)	1,700.170582	1,331.031627	360.6245005	46.13252132	4.751048729
E$_{2}$ (MPa)	1,700.170582	1,331.031627	360.6245005	46.13252132	4.751048729
E$_{a}$ (MPa)	1,699.997010	1,360.970271	1,002.429677	966.5762966	962.9909984
V$_{12}$	0.300130459	0.298320823	0.268011764	0.254911608	0.253220532
V13	0.300037122	0.286305936	0.106071965	0.032033916	0.022529523
V23	0.300037121	0.286305936	0.106071965	0.032033916	0.022529523
G$_{12}$ (MPa)	653.8461470	503.1115299	102.8961735	11.65915112	1.181960429
G$_{13}$ (MPa)	653.8461509	513.3786443	171.9071495	60.75475776	45.64068793
G$_{23}$ (MPa)	653.8461509	513.3786443	171.9071495	60.75475776	45.64068793

The engineering constants must be specified to ten significant figures to ensure that the material is fully described

### Kessler finite element model; varying tendon tissue properties

Increasing load was applied to the FE Kessler model, and the suture displacement was recorded. A comparison between experimental and FE results when tendon tissue material properties are varied are shown in Fig. 7. When employing a linear elastic isotropic material description of tendon, closest agreement with experimental results is achieved when Young’s modulus is 200 MPa and Poisson’s ratio is 0.4. The linear elastic orthotropic model agrees closest with experimental results when the young’s modulus of the fibrils is 1,700 MPa, the modulus of the matrix is between 10 and 1 MPa, and Poisson’s ratio for both is 0.3.

### Simulation of a 0.5 mm gap between tendon ends

**Fig. 8:**
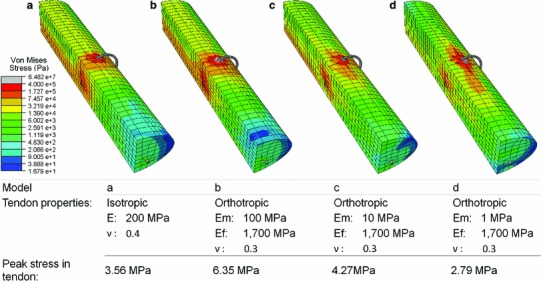
Deformation and stress map of Finite Element Kessler model when a 0.5 mm gap between the tendon ends is simulated. Tendon material description and peak stress in the tendon is detailed in the accompanying table. E = Young’s modulus, Em = modulus of the matrix, Ef = modulus of the fibrils, $\nu$ = Poisson’s ratio

Figure 8 shows the resultant deformation and stress map when simulating a 0.5 mm gap between the tendon ends in the Kessler repair. A high-stress region is observed around the grasping suture loops. As the modulus of the matrix is reduced and the material becomes more orthotropic, the high-stress region around the suture loop becomes more elongated along the length of the tendon.

**Fig. 9:**
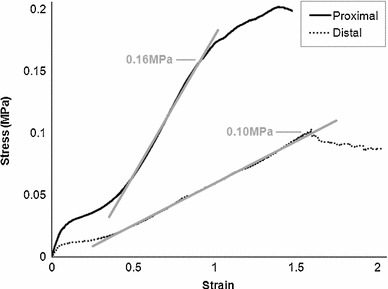
Mean stress-strain curves following transverse tensile testing of proximal and distal porcine FDP tendon showing peak stress beyond which the curves no longer present a linear relationship

We employ a linear elastic description for tendon behaviour, and it is therefore necessary to ensure this is an appropriate approximation for the tissue response within the range of deformation we apply to our model. Figure 9 shows the average stress-strain curves during transverse tensile testing of the porcine FDP tendon. The peak stress at which these curves behave linearly is 0.16 and 0.1 MPa for proximal and distal samples, respectively. From literature, the peak stress at which the human Achilles tendon, with a tangential modulus of 450 MPa, exhibits a linear relationship between stress and strain is 55 MPa (Lewis and Shaw 1997). These values indicate the peak stress, above which a linear elastic material assumption will not represent the tendon behaviour.

The isotropic material model employs a tangential modulus of 200 MPa, and a peak stress of 3.56 MPa is reached in the model (Fig. 8a), which is within acceptable limits for the linear material assumption when using longitudinal data (55 MPa). The orthotropic model is derived from the matrix and fibril modulus. Assuming a linear relationship between tangential modulus and peak stress, we deduce a peak stress for a linear assumption in the matrix and fibrils and then employ the rule of mixtures to deduce a peak stress for the homogenised material description employed in each analysis. The peak permissible stress is 156, 90, 84 and 83 MPa when the fibril modulus is 1,700 MPa and the matrix modulus is 1,000, 100, 10 and 1 MPa, respectively. The peak tendon stress in each model is within the limit of linear behaviour for each analysis performed (Fig. 8b to d)

## Discussion

The aim of this study was to observe the stress arising in the tendon tissue when load was applied to a sutured tendon repair. It was hypothesised that the high-stress region would agree with an elliptical area of acellularity present following suture repair and load application (Wong et al. 2010). The stress map resulting from isotropic material properties presents a circular high-stress region around the suture anchor (red and orange region on Fig. 8a). The high-stress region tends towards an elliptical shape as the microstructure matrix modulus tends towards 0.1 MPa when the orthotropic tendon description is employed (red and orange region on Fig. 8b to d). This supports the hypothesis that the acellular region arises due to high stress when suture and tensile loading are present (Wong et al. 2010). These results also highlight the importance of a suitable material description for tendon when employing FEA. In past sutured tendon analyses, an isotropic material description has been employed (Ingle et al. 2010; Funakoshi et al. 2008); however, results may differ had the highly orthotropic tendon properties been fully represented in the FE models. Simplifications are necessary during FE modelling, and we have employed several assumptions in our model. The suture knot was not modelled, yet this is often the failure location of suture repairs; however, our area of interest is stress in the tendon tissue, not the suture, which would not be affected by this simplification. We have also assumed perfect symmetry and perfect geometry within the repair, along with a perfectly transverse laceration, which is unlikely. It would be of interest to observe how varying the symmetry, geometry and angle of laceration affects stress patterns.

Our FE results show an augmented stress pattern due to the presence of the suture, including high stress around the anchor, and low stress by the tendon cut ends. It is well established that the application of load is beneficial to tendon healing, demonstrated by the improved strength and reduced restrictive adhesions following early active mobilisation, as opposed to passive mobilisation or immobilisation (Aoki et al. 1994; Wada et al. 2001; Halikis et al. 1997). In vitro tensile loading of stem-cell collagen sponge constructs results in significantly increased failure strength and stiffness compared with unloaded samples after 2 weeks (Juncosa-Melvin et al. 2006) demonstrating the benefits of axial tension. Additionally, the final stage of tendon healing involves realignment of collagen fibrils along the direction of loading which increases strength, thus the failure strength of the healing tissue approaches that of undamaged tendon (Woo et al. 1999). Under normal loading conditions, collagen aligns parallel to the long axis of the tendon (Galloway et al. 2013); however, the augmented stress pattern we observe when a Kessler suture is present (Fig. 8d) may affect realignment of the collagen. Realignment may be impaired in regions of stress shielding, as we observe near the tendon cut end, reducing localised tensile strength. Similarly, the direction of alignment may be altered due to the altered stress pattern throughout the suture site. Whilst an altered alignment of fibres may be suited to withstanding the altered loading environment, this may ultimately contribute to the inferior tensile strength observed in repaired tendon compared with healthy tendon (Burns et al. 2000), increasing the chance of long-term re-rupture.

Laboratory results during loading of a Kessler suture agree closest with isotropic FE results when the modulus is 200 MPa which was expected as this lies between the fibril and matrix modulus. When an orthotropic material description was employed, it was expected that FE results would tend towards laboratory results as the matrix modulus tended towards 0.1 MPa. Closest agreement is seen when matrix modulus is between 10 and 1 MPa. These unexpected results may arise from differences between the tendon microstructure and our idealised model. Assumptions were necessary to minimise computational cost. We have assumed the fibrils to be perfectly cylindrical, of regular cross sectional area, and in alignment with the long axis of the tendon. We did not account for the crimp pattern exhibited by fibrils (Rigby et al. 1959), irregularity and variation in fibril cross section (Starborg et al. 2013), or the effects of cells on the tissue, and we have not included a second level of tendon hierarchy. The impact on bulk properties when varying the microstructure geometry would be of interest to establish which microstructure features are essential and which are negligible when modelling tendon for this application.

Whilst we were able to perform FE analyses with properties approaching those found experimentally, it was not possible to obtain engineering constants when fibril modulus was 1,700 MPa and matrix modulus was 0.1 MPa, as measured in the tissue. During homogenisation, large strains would occur at the interface between the two severely different phases resulting in high distortion in the model which cannot be computed. In addition, Tendon is a viscoelastic material (Schwerdt et al. 1980); however, only the elastic behaviour has been accounted for in this model to minimise computing cost. Tendon also possesses a very high Poisson’s ratio which is over 0.8 (Cheng and Screen 2007; Lynch et al. 2003); however, Poisson’s ratio is limited to 0.5 when using Abaqus and a value close to 0.5 results in a material which is too stiff to sufficiently deform. The low Poisson’s ratio employed in this work is thought to have minimal impact on results as the tendon tissue is not excessively constrained. FEA does not provide exact results to a given problem, rather it provides an approximation. To obtain exact results an infinite number of elements would be necessary. As such, mesh effects may have contributed towards the difference between laboratory and expected FE results. Similarly, the homogenisation method does not provide an exact resultant bulk material description; however, it permits representation of the microstructure within the suture repair model, which we have seen impacts on the resultant stress pattern.

Ideally, the length of a tensile test sample would be at least 5 times the width. Sample length was limited by the tendon thickness, and difficulties in cutting a thin slice of fresh tendon using a scalpel. A desirable aspect ratio could have been achieved using a microtome as performed by Lake et al. (2010); however, this involves freezing the tendon which can impact on tendon mechanical properties (Clavert et al. 2001), thus we chose to test unfrozen tissue. Whilst the small aspect ratio we employed will cause error due to stress at the clamps, all samples failed at the mid-substance (Fig. 2) rather than the grip interface.

We have described a method of tendon modelling, suited to observing tendon stress resulting from suture repairs during loading, and demonstrated agreement between the high-stress region and acellularity in the tissue. Modelling alternative suture arrangements would permit comparison between acellular regions, without the expense and time required with animal models whilst also complying with the 3R’s (NC3Rs 2012). Clinically, this information would inform both suture selection and rehabilitation protocols. The model may also be adapted to analyse specific injuries, for example, obliquely lacerated tendons or those exhibiting tissue degradation as seen in spontaneous rupture (Kannus and Józsa 1991).
